# Inflammasome-associated immune dysregulation in TB–HIV coinfection

**DOI:** 10.3389/fcimb.2026.1961755

**Published:** 2026-09-30

**Authors:** Nathalia Beatriz Ramos de Sá, Mariana Rosa Inacio Coelho, Marcelo Ribeiro-Alves, Dalziza Victalina de Almeida, Karine Venegas Macieira, Daniela Machado de Luca, Diogo Gama Caetano, Tatiana Pereira da Silva, José Henrique Pilotto, Valeria Cavalcanti Rolla, Carmem B. W. Giacoia-Gripp, Daniel Scott-Algara, Sylvia Lopes Maia Teixeira, Mariza Gonçalves Morgado

**Affiliations:** 1 Fundação Oswaldo Cruz, Fiocruz, Instituto Oswaldo Cruz (IOC), Laboratório de AIDS e Imunologia Molecular, Rio de Janeiro, Brazil; 2 Fundação Oswaldo Cruz, Fiocruz, Instituto Nacional de Infectologia Evandro Chagas (INI), Laboratório de Pesquisa Clínica em Micobacterioses, Rio de Janeiro, Brazil; 3 Nova Iguaçu General Hospital, Nova Iguaçu, Rio de Janeiro, Brazil; 4 Direction International, Institut Pasteur, Paris, France

**Keywords:** HIV-1, inflammasome, TB-HIV co-infection, TB-HIV/IRIS, tuberculosis

## Abstract

**Background:**

Tuberculosis (TB) and HIV co-infection are characterized by dysregulated inflammation and increased risk of immune reconstitution inflammatory syndrome (IRIS) following antiretroviral therapy (ART) initiation. Inflammasomes are key regulators of innate immune responses, but their role in TB–HIV co-infection and IRIS remains incompletely understood. We investigated inflammasome-related gene expression and cytokine profiles in TB, HIV-1, and TB–HIV individuals, and their association with IRIS development.

**Methods:**

Fifty-nine participants were stratified into four groups: TB–HIV (n = 20; 4 with IRIS), HIV-1 (n = 12), TB (n = 17), and healthy controls (n = 10). These individuals were followed up at HGNI and INI-Fiocruz (Rio de Janeiro/Brazil) from 2014 to 2016. Whole-blood PAXgene expression of inflammasome-related genes (AIM2, CASP1, GSDMD, IL1β, IL18, and NLRP3) was quantified using TaqMan RT–qPCR. Plasma levels of IL-1β, IL-18, IL-33, and IL-6 were measured by ELISA. Associations were evaluated using multivariable linearregression and partial Pearson correlation analyses.

**Results:**

TB–HIV co-infected individuals showed significantly higher expression of AIM2, CASP1, GSDMD, IL1β, and IL18 compared to all other groups (all P < 0.01), while NLRP3 expression remained unchanged. Elevated plasma levels of IL-6, IL-18, and IL-33 further supported a systemic inflammatory profile in the TB-HIV co-infection group. Strong positive correlations were observed among AIM2, CASP1, GSDMD, IL1β, and IL18 gene expressions at baseline (r= 0.853–0.965), and, to a lesser extent, among them and IL-6 and IL-18 cytokines (r= 0.440-0.645), indicating a coordinated inflammasome-related transcriptional profile, whereas NLRP3 showed no or weak associations. Longitudinal analysis revealed significant reductions in AIM2, CASP1, GSDMD, IL18, and IL1B expression from baseline to week 2, after two weeks of anti-TB therapy and before ART initiation, and from baseline to week 6, after six weeks of anti-TB therapy and four weeks of ART (all P < 0.05).

**Conclusions:**

TB–HIV coinfection was characterized by distinct inflammasome-related transcriptional and inflammatory profiles, highlighting AIM2 as a potential pathway for further investigation. Our findings indicate that TB–HIV coinfection is associated with a coordinated inflammasome-related transcriptional and cytokine profile, characterized by increased expression of AIM2 and downstream effector genes, without detectable changes in NLRP3 transcript abundance.

## 1 Introduction

Tuberculosis (TB) and AIDS remain the major causes of death from infectious diseases worldwide. Despite major progress in reducing the global number of new HIV infections (≈ 40% compared to 1997) through the development of potent antituberculosis and antiretroviral drug combinations and the widespread implementation, both diseases remain at high levels, mainly in high-risk populations (Global Tuberculosis Report 2025, 2025).

Both diseases are characterized by dysregulated inflammatory responses, which are further amplified in individuals with TB–HIV coinfection. Inflammation is one major mechanism of innate immunity in this process. Among other mechanisms, this inflammatory response can be mediated by large protein complexes called inflammasomes located in the cytoplasm. The inflammasome unit is composed of a sensor protein, an adaptor, and a procaspase protein that assemble in the cytosol after sensing pathogen-associated molecular patterns (PAMPs) or damage-associated molecular patterns (DAMPs) (Lamkanfi and Dixit, 2014; Sharma and Kanneganti, 2016). Major inflammasome sensor proteins include NLRP1, NLRP3, NLRP6, AIM2, Pyrin, and CARD8, among others, each recognizing distinct microbial or endogenous danger signals (Fu et al., 2024; Dubey et al., 2025). Once activated, inflammasome sensor proteins recruit the adaptor ASC and procaspase-1 to assemble the inflammasome complex. Upon activation, canonical inflammasome sensors oligomerize and recruit procaspase-1, either directly or through the adaptor ASC, thereby enabling caspase-1 activation. Active caspase-1 cleaves pro-IL-1β and pro-IL-18 into their mature forms and processes GSDMD, whose N-terminal fragment forms membrane pores that promote cytokine release and pyroptotic cell death, thereby amplifying inflammation (Barnett et al., 2023; Coll and Schroder, 2024). Persistent inflammasome activation may maintain immune cells in a chronically hyperactivated state, thereby contributing to sustained inflammation (Dubey et al., 2025).

Accumulating evidence has demonstrated the involvement of inflammasomes in both HIV and simian immunodeficiency virus (SIV) infections, where persistent inflammasome activation contributes to chronic immune activation and disease progression (Wang and Shan, 2022; Zhang et al., 2025). The progressive CD4^+^ T-cell depletion observed in pathogenic infections has been related to the activation of the inflammasome (in particular CARD8) following virus entry into susceptible cells (Wang et al., 2024), which is not observed in non-pathogenic models. Despite major advances in antiretroviral therapy and the management of HIV-associated co-infections, persistent immune activation and chronic inflammation remain hallmarks of HIV infection, even among individuals who control HIV replication (Deeks et al., 2015). The level of inflammation is often associated with, and predictive of, HIV disease progression. This inflammation could be worsened by other inflammatory comorbidities, in particular TB. TB clinical manifestations in patients with HIV/AIDS are much more severe than in immunocompetent individuals. They are characterized by extrapulmonary and miliary or disseminated presentations and worsening immunosuppression, reducing the ability of granulomas to contain the bacilli, leading to their multiplication and release, inducing mycobacteremia, culminating in severe clinical condition and severe pathology with a significant increase in TB-related mortality (Schmaltz et al., 2009; Shankar et al., 2014). Although the use of antiretroviral therapy *(*ART) during TB treatment improves patient survival, particularly by reducing viral load and restoring immune function, the coadministration of drugs is challenging due to drug to drug interactions and/or the onset of clinical and radiological worsening resulting from an exacerbated inflammatory reaction as a consequence of immune reconstitution, known as immune reconstitution inflammatory syndrome (TB-HIV/IRIS) (Meintjes et al., 2008).

In this regard, some investigations have found that TB-HIV/IRIS is associated with changes in the expression of cytokines related to the inflammasome activation pathway, and other proinflammatory cytokines, such as IL-1β, IL-18, IL-33, IL-6, IL-17, IL-22, TNF, and IFN-γ, supporting their role in the development of TB-HIV/IRIS (Tadokera et al., 2011; Conesa-Botella et al., 2012; Tan et al., 2015; Tan et al., 2016; Ravimohan et al., 2018). We previously reported that polymorphisms in genes encoding the inflammasome complex could be associated with the clinical presentation of TB in HIV infected individuals, as well as IRIS, and secretion of cytokines (Sá et al., 2022). Recent reports seem to confirm the role of the inflammasome complex in the pathogenesis of HIV infection (Zhang et al., 2024; Pan et al., 2025) and point out the multiple inflammasome activators that emerged during HIV infection (Zhang et al., 2025).

In the present longitudinal study, we hypothesized that TB–HIV coinfection would be associated with an altered inflammasome-related transcriptional and cytokine profile and that these markers would change during anti-TB therapy and subsequent ART. To address this hypothesis, we assessed the mRNA expression of inflammasome-related genes (AIM2, CASP1, GSDMD, IL18, IL-1β, and NLRP3) and plasma proinflammatory cytokines. We further investigated their potential association with susceptibility to TB and/or HIV-1, their longitudinal dynamics following the sequential initiation of anti-TB therapy and ART, and their relationship with the development of TB-HIV/IRIS.

## 2 Materials and methods

### 2.1 Patient enrolment and study design

This study was nested within a previously described genetic, clinical, and immunological longitudinal study conducted at the Laboratory of AIDS and Molecular Immunology (IOC/FIOCRUZ) between 2014 and 2016 (Giacoia-Gripp et al., 2019; de Sá et al., 2020; Sá et al., 2022). All participants signed an informed consent form, and the local ethics committee approved the studies.

A total of 59 participants were stratified into four groups: individuals co-infected with TB and HIV-1 (TB–HIV group, n = 20; including 04 who developed paradoxical TB-associated immune reconstitution inflammatory syndrome [TB-HIV/IRIS]); individuals living with HIV-1 without TB (HIV-1 group, n = 12); individuals with TB who were HIV-1 seronegative (TB group, n = 17); and healthy controls without TB or HIV-1 infection (HC group, n = 10).

The individuals were enrolled and followed up at the Clinical Research Laboratory on Mycobacteria (LAPCLINTB) of the National Institute of Infectious Diseases Evandro Chagas, Oswaldo Cruz Foundation (INI/FIOCRUZ), Rio de Janeiro, Brazil and at the Nova Iguaçu General Hospital (HGNI), Rio de Janeiro, Brazil, from 2014 to 2016. Detailed information on eligibility criteria, treatment regimens (anti-TB and combination antiretroviral therapy), study design, demographic and clinical data at the study entry visit, and sample availability has been previously described (Giacoia-Gripp et al., 2019). Absolute and percentage CD4 and CD8 values, as well as viral load data, were retrieved from the medical records of participants in the HIV and TB–HIV groups at the two clinical sites participating in this study. All TB-HIV coinfected individuals were prospectively evaluated at the introduction of anti-TB therapy at the baseline visit (D0), at the initiation of ART (W2), and during the clinical follow- up visits at weeks 6, 10, 14, and 24 to assess the development of TB-HIV/IRIS.

All IRIS cases were classified as paradoxical TB-HIV/IRIS, defined as a worsening of TB signs and symptoms starting after ART initiation during TB treatment, mainly presenting enlargement of lymph nodes and inflammatory signs, not explained by any other diseases or by an adverse effect of drug therapy (Robertson et al., 2006; Meintjes et al., 2008), as previously detailed by our group (Demitto et al., 2019). In general, the IRIS cases included in the present study were self-resolving; when required, patients received corticosteroid therapy (e.g., prednisone).

Self-reported skin color was classified according to the criteria established by the Brazilian Institute of Geography and Statistics (IBGE) (Instituto Brasileiro de Geografia e Estatística, 2013).

### 2.2 Blood collection and nucleic acid isolation

Whole blood was collected in PAXgene Blood RNA Tubes (PreAnalytiX, Qiagen) at three time points and stored at −80 °C until processing: at baseline (D0), immediately before the first dose of anti-TB therapy and prior to ART initiation; at week 2 (W2), after two weeks of anti-TB treatment and immediately before the first dose of ART; and at week 6 (W6), after six weeks of anti-TB therapy and four weeks of ART. Total RNA was extracted using the PAXgene Blood RNA Kit (Qiagen) following the manufacturer’s protocol. Briefly, samples were centrifuged, lysed, and treated with proteinase K before homogenization using PAXgene Shredder columns. After ethanol addition, lysates were transferred to spin columns for RNA binding, followed by wash steps and DNase I treatment. RNA was eluted and heat-denatured at 65 °C for 2 min.

RNA concentration and purity were assessed using a NanoDrop 8000 spectrophotometer, whereas RNA integrity was evaluated using an Agilent 4200 TapeStation system (Agilent Technologies Inc., Santa Clara, CA, USA). Only samples with an RNA integrity number (RIN) greater than 7.0 were used for further analysis.

### 2.3 cDNA synthesis and relative quantification of AIM2, CASP1, GSDMD, IL18, IL-1β, and NLRP3 gene expression

Complementary DNA (cDNA) was synthesized from total RNA using M-MLV reverse transcriptase (Invitrogen), according to the manufacturer’s instructions, and stored at −20 °C.

Gene expression was quantified by real-time PCR using the commercial TaqMan Gene Expression Assays (Applied Biosystems, Foster City, CA) for AIM2, CASP1, GSDMD, IL18, IL-1β, and NLRP3 (**Supplementary Table S1**). The β-actin (ACTB), glyceraldehyde-3-phosphate dehydrogenase (GAPDH), and 18S ribosomal RNA (18S) genes were used as housekeeping genes to normalize the gene expression. Thermal cycling conditions were as follows: 50 °C for 2 min, 95 °C for 10 min, followed by 40 cycles of 95 °C for 15 s and 60 °C for 1 min. Negative controls were included in all runs. Amplifications were performed on the ABI7500 Real-Time platform (Applied Biosystems/AB and Life Technologies), available at the Fiocruz Real-Time PCR Platform.

### 2.4 Plasma collection and cytokine measurement

Plasma samples were available for cytokine measurement at baseline (D0) and week 2 (W2). Plasma concentrations of the pro-inflammatory cytokines included in the study were measured at the enrolment D0 and W2, as follows: IL-1b/IL-1F2 (DuoSet ELISA Kit, R & D Systems, #DY201); IL-18 (Human Instant ELISA Kit, Thermo Fisher, BMS267INST); and IL-6 and IL-33 (Human Mini ABTS ELISA Development Kit, PeproTech, Inc., Rocky Hill, NJ) according to the manufacturer’s instructions. These data have been partially described (Sá et al., 2022).

### 2.5 Statistical analyses

Descriptive statistics were calculated to summarize the sociodemographic, clinical, and laboratory characteristics of the study participants. Continuous variables were expressed as medians and interquartile ranges (IQR), while categorical variables were presented as absolute and relative frequencies. To evaluate differences at baseline among the four study groups (healthy controls, HIV, TB, and TB–HIV), chi-square tests were applied for nominal variables. For continuous numerical variables, Kruskal-Wallis H tests were utilized for comparisons involving three or more groups (e.g., the four study groups), and Mann-Whitney U tests were used for two-group comparisons. To meet model assumptions and approximate normality, raw cytokine concentrations and laboratory counts (IL-1β, IL-6, IL-18, IL-33, CD4, CD8, CD4/CD8 ratio, and viral load) were transformed using the formula Log10(x+10)-1, while gene expression values (AIM2, CASP1, GSDMD, IL18, IL1β, and NLRP3) were transformed using the formula Log2(x+2)-1.

Cross-sectional comparisons of transformed molecular markers among the four study groups at baseline were performed using multivariable ordinary least squares (OLS) linear regression models (`lm` function, R `stats` package). These models included the study group as the independent variable and were adjusted for *a priori* selected confounders: age, gender, self-reported skin color (dichotomized for statistical modeling as white vs. brown/black), and educational level (dichotomized as high vs. low). For specific pairwise group comparisons (e.g., TB–HIV vs. TB, and TB–HIV vs. HIV), baseline CD4+ T-cell counts were additionally included as a confounder in the OLS models. Estimated marginal means and pairwise contrasts between groups were derived using the `emmeans` package, with p-values adjusted for multiple comparisons using the Tukey method.

To assess the association between molecular markers and the development of IRIS, OLS linear regression models were applied to the baseline data of the TB–HIV subgroup, comparing IRIS and non-IRIS individuals while adjusting for age, gender, skin color, and schooling.

The relationships among gene expression markers and plasma cytokines were evaluated using partial Pearson correlation coefficients. Briefly, confounding variables (age, gender, skin color, and schooling) were regressed out from each molecular marker using OLS, and pairwise Pearson correlations were then computed on the resulting residuals (`Hmisc` and `corrplot` packages). Only statistically significant correlations (P < 0.05) were retained and visualized.

An exploratory principal component analysis (PCA) was conducted on the baseline log-transformed molecular data (10 markers) using the `PCA` function from the `FactoMineR` package. Before PCA, the data were standardized to z-scores, and listwise deletion was applied for missing values. The resulting principal components were visualized using biplots generated with the `factoextra` package.

Finally, a longitudinal variation in gene expression and cytokine levels within the TB–HIV group across the three clinical visits (D0, W2, and W6) was evaluated using linear mixed-effects models (LME) via the `lmer` function (R `lme4` package). The models included the timepoint (Visit) as a fixed effect, adjusted for age, gender, skin color, schooling, and CD4+ T-cell count, while incorporating a random intercept for each participant to account for within-subject repeated measures. *Post-hoc* pairwise contrasts between timepoints were estimated using marginal means with Tukey’s adjustment.

All analyses were conducted in R software (version 4.1.1).

## 3 Results

### 3.1 Participant characteristics

The sociodemographic, clinical, and laboratory characteristics of the 59 participants at study entry, stratified by study group (healthy controls, HIV, TB, and TB–HIV), are presented in **Table 1**. Among the 20 TB–HIV co-infected individuals, four developed paradoxical TB-HIV/IRIS. Most participants were male (64.4%), with no significant differences across groups (P = 0.222). Self-reported skin color distribution was comparable between white (33.9%) and brown (50.8%) individuals, whereas black individuals comprised 15.3% of the cohort. Regarding educational levels, 50.8% of the individuals had lower secondary education, and 30.5% had an upper secondary education. Significant differences in CD4+ T lymphocyte counts (P<0.001) and the CD4/CD8 ratio (P<0.001) were observed between groups, with markedly reduced levels in HIV-1 and TB–HIV groups, as expected. In contrast, absolute CD8^+^ T cell counts did not differ across groups (P = 0.988).

**Table 1:** Sociodemographic, clinical, and laboratory characteristics of the individuals included in the study at baseline.

Features	Levels	Overall	Groups				P-value^a^
			HC (n=10)	HIV (n=12)	TB (n=17)	TB-HIV (n=20)	
Sex	Male	38 (64.4%)	4 (40%)	9 (75%)	10 (58.8%)	15 (75%)	0.222
	Female	21 (35.6%)	6 (60%)	3 (25%)	7 (41.2%)	5 (25%)	
Skin Color^b^	Brown	30 (50.8%)	4 (40%)	8 (66.7%)	7 (41.2%)	11 (55%)	0.35
	White	20 (33.9%)	5 (50%)	4 (33.3%)	7 (41.2%)	4 (20%)	
	Black	9 (15.3%)	1 (10%)	0 (0%)	3 (17.6%)	5 (25%)	
Schooling^c^	Primary education	3 (5.1%)	0 (0%)	0 (0%)	2 (11.8%)	1 (5%)	0.323
	Lower secondary education	30 (50.8%)	4 (40%)	6 (50%)	8 (47.1%)	12 (60%)	
	Upper secondary	18 (30.5%)	3 (30%)	5 (41.7%)	4 (23.5%)	6 (30%)	
	Bachelor	6 (10.2%)	3 (30%)	1 (8.3%)	1 (5.9%)	1 (5%)	
	Unknown	2 (3.4%)	0 (0%)	0 (0%)	2 (11.8%)	0 (0%)	
Age, years	Age, years	37 (IQR = 11)	30.5 (IQR = 14)	32.5 (IQR = 11.25)	42 (IQR = 10)	36 (IQR = 7.25)	0.257
CD4 count (cells/µL); n		176 (IQR = 744)	1084.5 (IQR = 448)	133.5 (IQR = 108)	745 (IQR = 351)	54.5 (IQR = 119.25)	**< 0.001**
CD8 count (cells/µL); n (%)		521 (IQR = 499.5)	580.5 (IQR = 300.75)	579.5 (IQR = 559.25)	453 (IQR = 390)	482 (IQR = 578)	0.988
HIV-1 viral load (VL), copies/mL^d^	HIV-1 viral load, copies/mL	390,479.5 (IQR = 601,338.5)	not applicable	237,389.5 (IQR = 210,014.25)	not applicable	664,127.5 (IQR = 720,224.25)	**0.022**
Log_10_ HIV-1 viral load	Log_10_ HIV-1 viral load	5.59 (IQR = 0.66)	not applicable	5.37 (IQR = 0.38)	not applicable	5.82 (IQR = 0.58)	**0.022**
CD4 count (cells/µL)	≤ 200 cells/µL	33 (55.9%)	0 (0%)	12 (100%)	1 (5.9%)	20 (100%)	**< 0.001**
	> 200 cells/µL	26 (44.1%)	10 (100%)	0 (0%)	16 (94.1%)	0 (0%)	
CD8 count (cells/µL)	≤ 500 cells/µL	28 (47.5%)	3 (30%)	5 (41.7%)	10 (58.8%)	10 (50%)	0.509
	> 500 cells/µL	31 (52.5%)	7 (70%)	7 (58.3%)	7 (41.2%)	10 (50%)	
CD4/CD8 ratio	≤ 1	38 (64.4%)	1 (10%)	12 (100%)	5 (29.4%)	20 (100%)	**< 0.001**
	>1	21 (35.6%)	9 (90%)	0 (0%)	12 (70.6%)	0 (0%)	

^a^ P-values were calculated using chi-square tests for nominal variables, and Kruskal-Wallis H tests for continuous numerical variables. Associations were considered significant at P < 0.05. ^b^Skin color categorization followed the classificatory system employed by the Brazilian Institute of Geography and Statistics (IBGE) (Instituto Brasileiro de Geografia e Estatística, 2013). ^c^Classification, according to the International Standard Classification of Education (ISCED) maintained by the United Nations Educational, Scientific and Cultural Organization (UNESCO). ^d^ HIV-1 viral load was available only for participants in the HIV and TB–HIV groups. Viral load was not applicable to the healthy control and TB groups. For statistical modeling of p-values, self-reported skin color was dichotomized into “white” and “brown/black” categories, and educational level was dichotomized into “high” (Upper secondary and Bachelor) and “low” (Primary education, Lower secondary education, and unknown). HC, healthy controls; N, number of individuals in each group; TB, tuberculosis; %, frequencies; IQR, interquartile range. Bold values indicate statistically significant P-values (P < 0.05).

### 3.2 Inflammasome-related gene expression and plasma cytokines

Relative baseline mRNA expression levels of AIM2, CASP1, GSDMD, IL18, IL-1β, and NLRP3, and plasma levels of cytokines IL-1β, IL-6, IL-18, and IL-33 across study groups are shown in **Figure 1**. All cross-sectional group comparisons were adjusted for age, gender, self-reported skin color, and educational level; pairwise comparisons involving the TB-HIV group were additionally adjusted for baseline CD4+ T-cell counts to account for differences in disease severity.

**Figure 1:**
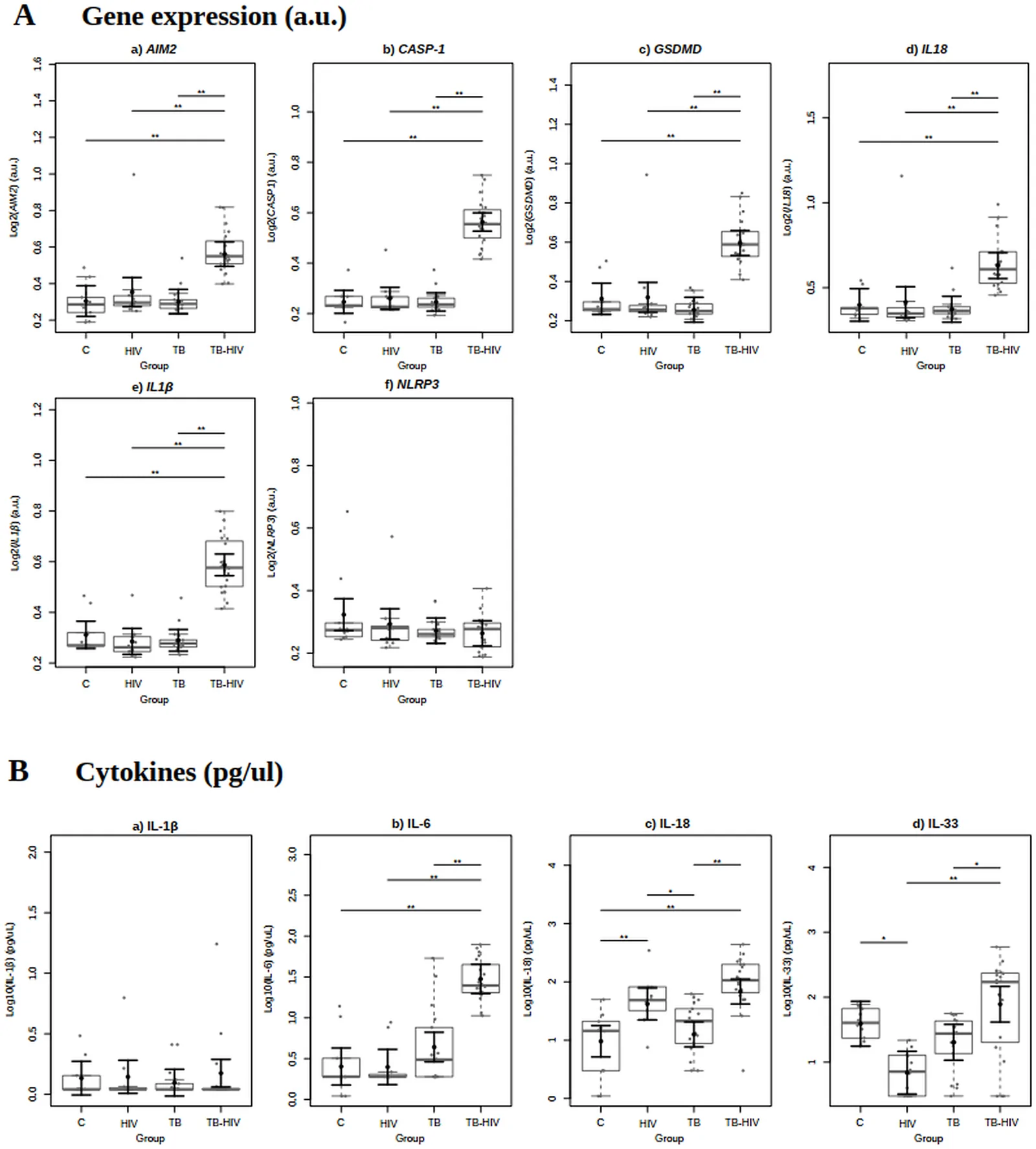
Comparison of whole-blood inflammasome-related gene expression **(A)** and plasma cytokine levels **(B)** among healthy controls **(C)**, HIV group (HIV), TB group (TB), and TB–HIV group at baseline. **(A)** AIM2 **(a)**, CASP1 **(b)**, GSDMD **(c)**, IL18 **(d)**, IL1β **(e)**, and NLRP3 **(f)** mRNA expressions. **(B)** Plasma levels of IL-1β **(a)**, IL-6 **(b)**, IL-18 **(c)**, and IL-33 **(d)**. Boxplots show the distribution of log-transformed values within each group. Boxes represent the interquartile range (IQR), and the central horizontal bars represent the sample medians. Highlighted black vertical lines represent the 95% confidence intervals (95% CI) of the estimated marginal means derived from multivariable ordinary least squares linear regression models adjusted for age, gender, skin color, and schooling (the central circle indicates the marginal mean). Gene expression values were transformed using Log*_2_(x+2)-1*, and cytokine concentrations were transformed using *Log_10_(x+10)-1*. Pairwise contrasts between marginal means were estimated with Tukey’s adjustment. *P < 0.05; **P < 0.01.

TB–HIV co-infected individuals showed significantly higher expression of AIM2, CASP1, GSDMD, IL-18, and IL-1β, but not NLRP3, compared with the other groups. Compared with the TB group, AIM2, CASP1, GSDMD, IL-18, and IL-1β expression levels were increased by approximately 20%, 25%, 27%, 20%, and 23%, respectively (Log_2_FC range: 0.2570–0.3397; all P < 0.0001). Compared with the HIV group, the corresponding increases were approximately 16%, 23%, 21%, 16%, and 23%, respectively (Log_2_FC range: 0.2079–0.3029; all P ≤ 0.0016). Similarly, relative to the healthy control group, expression levels were elevated by approximately 19%, 25%, 22%, 17%, and 21%, respectively (Log_2_FC range: 0.2315–0.3167; all P ≤ 0.0026). No significant differences in NLRP3 mRNA expression were observed (**Figure 1A**).

Consistent with these findings, plasma levels of IL-6, IL-18, and IL-33 were significantly higher in individuals with TB–HIV co-infection compared to those in the TB group (all P < 0.0136). Similarly, levels of IL-6 and IL-33 were markedly higher in individuals with TB-HIV than in the HIV group. (all P < 0.0001). In addition, IL-6 and IL-18 levels were higher in TB–HIV individuals compared to healthy controls group (all P < 0.0001). Among the HIV group, IL-18 levels were increased compared to both healthy controls group (P = 0.0083) and the TB group (P = 0.0198), whereas IL-33 levels were lower than those observed in healthy controls group (P = 0.0143). No significant differences in plasma levels of IL-1β were observed (**Figure 1B**).

Exploratory analysis were also conducted comparing individuals with and without IRIS, by both gene expression (A) and cytokine analyses (B), and only plasma IL-1β levels showed a statistically significant difference between the groups (P = 0.0203) (**Figure 2**).

**Figure 2:**
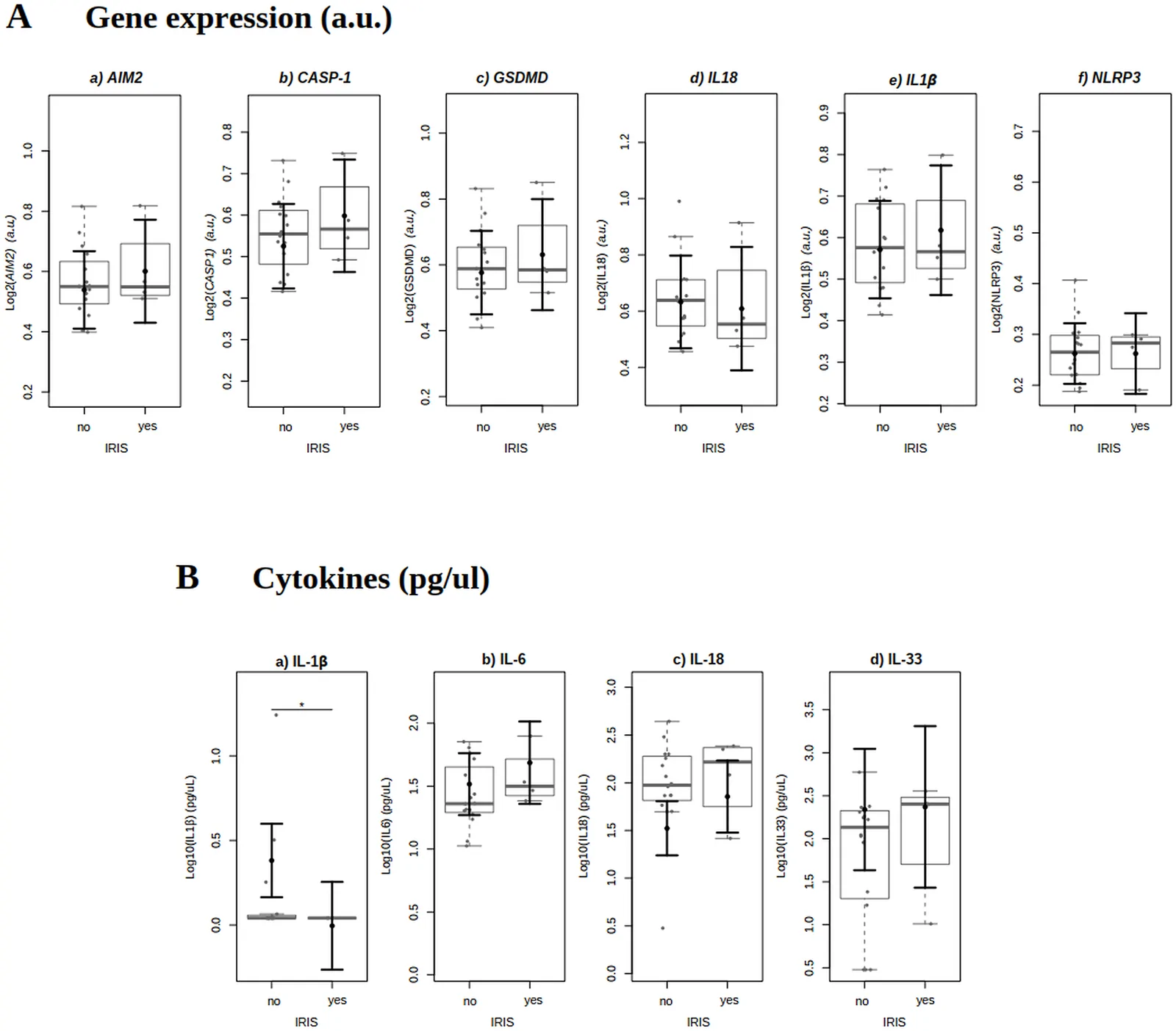
Comparison ofwhole-blood inflammasome-related gene expression **(A)** and plasma cytokine levels **(B)** between TB–HIV individuals with and without IRIS at baseline. **(A)** AIM2 **(a)**, CASP1 **(b)**, GSDMD **(c)**, IL18 **(d)**, IL1β **(e)**, and NLRP3 **(f)**. **(B)** Plasma levels of IL-1β **(a)**, IL-6 **(b)**, IL-18 **(c)**, and IL-33 **(d)**. Boxplots show the distribution of log-transformed values within each group. Boxes represent the interquartile range (IQR), and the central horizontal bars represent the sample medians. Highlighted black vertical lines represent the 95% confidence intervals (95% CI) of the estimated marginal means derived from multivariable ordinary least squares linear regression models adjusted for age, gender, skin color, and schooling (the central circle indicates the marginal mean). Gene expression values were transformed using Log2(x+2)-1, and cytokine concentrations were transformed using Log10(x+10)-1. Pairwise contrasts between marginal means were estimated withTukey’s adjustment. P < 0.05.

No significant differences related to inflammasome-related mRNA expressions or proinflammatory plasma cytokines were found between individuals with pulmonary and extrapulmonary TB in individuals with TB–HIV co-infection (data not shown).

### 3.3 Correlations between the inflammasome-related gene expression and plasma cytokines

**Figure 3** presents the Pearson correlation matrix for all evaluated markers measured in individuals from the TB–HIV, HIV, TB, and healthy control (HC) groups, allowing the assessment of potential associations among inflammasome-related genes and inflammatory mediators across the study population. Strong positive correlations were observed among inflammasome-related gene expression markers, particularly between AIM2, CASP1, GSDMD, IL-1β, and IL-18 (r = 0.853–0.965), indicating a highly coordinated inflammasome-related transcriptional profile. Notably, CASP1 correlated strongly with IL-1β (r = 0.949, P < 0.0001), GSDMD (r = 0.895, P < 0.0001), and AIM2 (r = 0.853, P < 0.0001), while GSDMD also showed strong correlations with IL-18 (r = 0.935, P < 0.0001) and IL-1β (r = 0.915, P < 0.0001). AIM2 expression demonstrated strong correlations with GSDMD (r = 0.943, P < 0.0001), IL-18 (r = 0.965, P < 0.0001), and IL-1β (r = 0.884, P < 0.0001), and moderate correlations with cytokines, including IL-6 (r = 0.440, P < 0.001) and plasma IL-18 (r = 0.561, P < 0.0001), highlighting the strong association of AIM2 expression with other inflammasome-related molecules. Moderate positive correlations were also observed between circulating cytokines and gene expression markers. IL-6 showed moderate correlations with CASP1 (r = 0.645, P < 0.0001), IL-1β (r = 0.610, P < 0.0001), and GSDMD (r = 0.517, P < 0.0001), while plasma IL-18 levels correlated moderately with AIM2 (r = 0.561, P < 0.0001), CASP1 (r = 0.556, P < 0.0001), and GSDMD (r = 0.562, P < 0.0001). In contrast, NLRP3, which displayed very low mRNA expression levels across the studied groups, showed only weak to moderate correlations with the other markers (r = 0.116–0.438). Overall, this correlation pattern was characterized by strong associations among AIM2-related transcriptional markers, whereas NLRP3 showed comparatively weaker associations with other inflammasome evaluated molecules (**Figure 3**).

**Figure 3:**
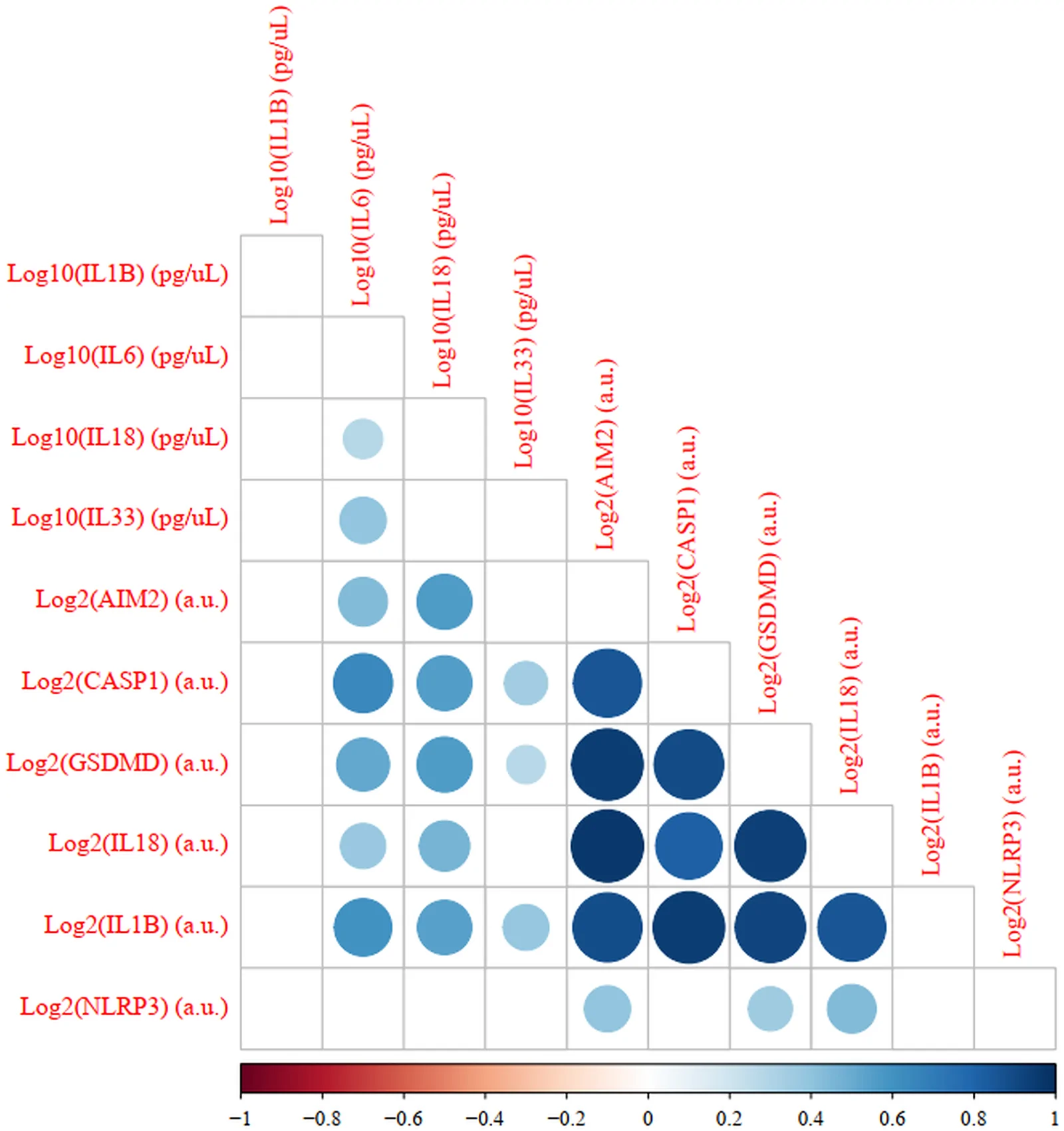
Partial Pearson correlation matrix showing associations between whole-blood inflammasome-related gene expression markers and plasma cytokine levels at baseline. Circle size and color intensity correspond to the magnitude of the partial correlation coefficient (r). Correlations were calculated on the residuals of linear models regressing out age, gender, skin color, and schooling. Only statistically significant correlations (P < 0.05) are displayed.

### 3.4 Principal component analysis

Principal component analysis (PCA) was performed using inflammasome-related gene expression markers and plasma cytokine levels to explore the overall inflammatory profile across the four study groups (**Figure 4**). The first two principal components explained 71.6% of the total variance. The first component (Dim1) represented 57.9% of the total variance, partially separating TB–HIV co-infected individuals from the other groups, suggesting an exploratory trend toward a distinct inflammatory signature associated with coinfection while the second (Dim2) represented 13.7% of the total variance. Inflammasome-related variables, particularly AIM2, CASP1, GSDMD, IL18, and IL1B, contributed most strongly to group separation, whereas NLRP3 showed a comparatively lower contribution. Notably, almost all individuals from the healthy control (C), HIV, and TB groups were concentrated in the quadrants to the left of the origin (second and third quadrants), indicating similar expression profiles among them for these specific variables. The TB-HIV group, in turn, exhibited greater dispersion, though it was predominantly concentrated on the right side of the biplot (first and fourth quadrants), coinciding with the higher contribution of the inflammasome gene expression variables (**Figure 4**).

**Figure 4:**
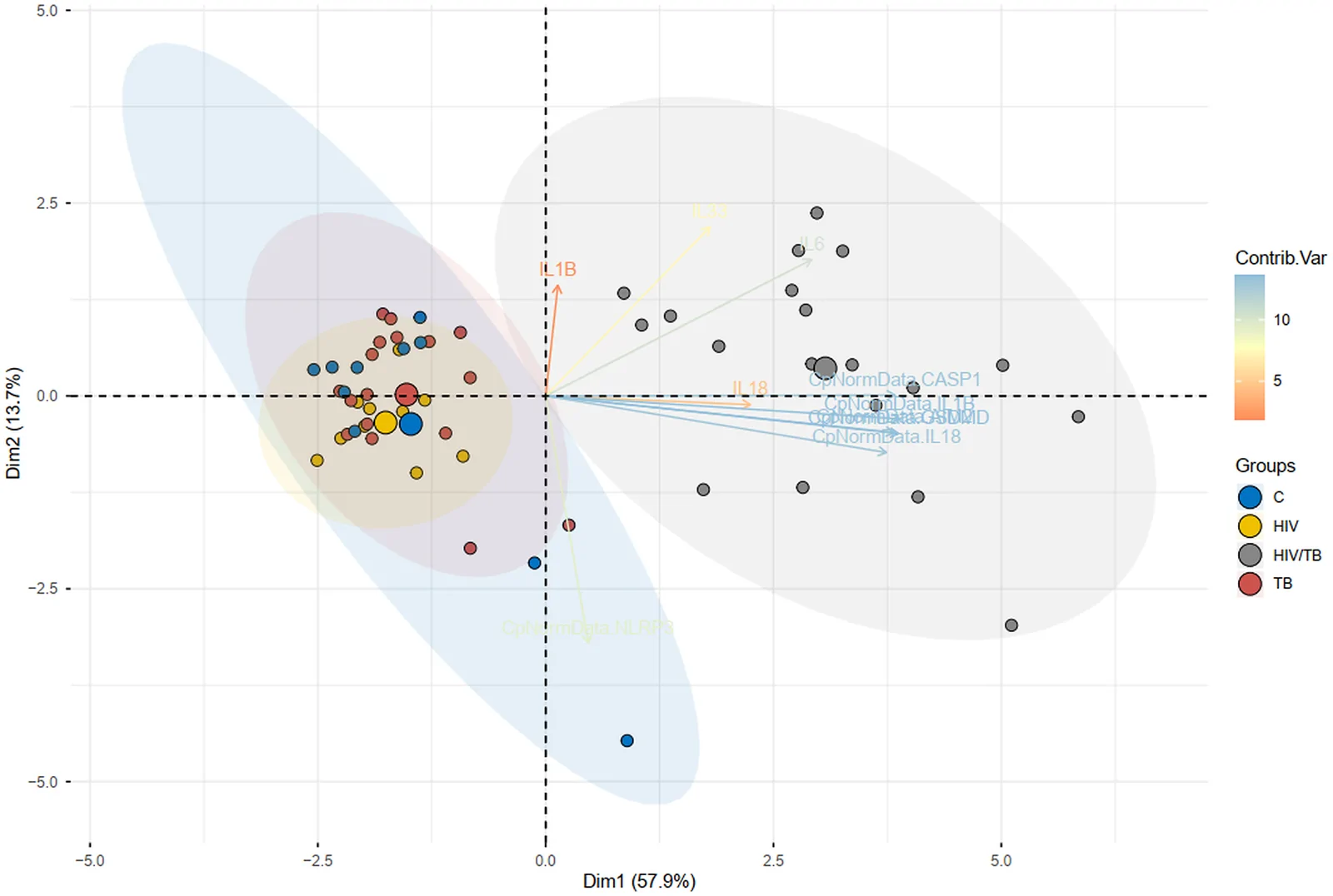
Principal component analysis (PCA) biplot showing the distribution of baseline samples across the first two principal components (Dim 1 and Dim 2). The circles representing each sample are colored according to study groups (C, HIV, TB, and TB-HIV), with confidence ellipses plotted for each group. The arrows represent the loading vectors of each evaluated molecular marker, with colors indicating their relative contribution to the principal components (gradient scale from low to high contribution). Data were standardized to z-scores before PCA.

### 3.5 Longitudinal analysis of the inflammasome-related gene expression in response to the anti-tuberculosis treatment and ART initiation in the TB-HIV group.

Longitudinal analysis showed significant modulation of inflammasome-related gene expression (AIM2, CASP1, GSDMD, IL-18, and IL-1β) throughout the clinical follow-up of TB-HIV individuals. Two weeks after anti-TB treatment initiation and before ART initiation (week 2), mRNA expression levels of AIM2, CASP1, GSDMD, IL-18, and IL-1β were reduced by approximately 11%, 10%, 10%, 10%, and 11%, respectively (Log_2_FC range: −0.1734 to −0.1502; all P ≤ 0.0255), outlining the possible results of the anti-TB treatment. Further reductions were observed in week 6, after six weeks of anti-TB therapy and four weeks of ART, with decreases of approximately 12%, 11%, 15%, 11%, and 14%, respectively (Log_2_FC range: −0.2379 to −0.1609; all P ≤ 0.0491). No significant differences in NLRP3 expression were observed across the evaluated time points (**Figure 5A**). Regarding cytokines, only IL-6 showed significant modulation during follow-up, with a significant decrease observed after two weeks of anti-TB treatment and before ART initiation (week 2) compared to baseline (P<0.0001) (**Figure 5B**).

**Figure 5:**
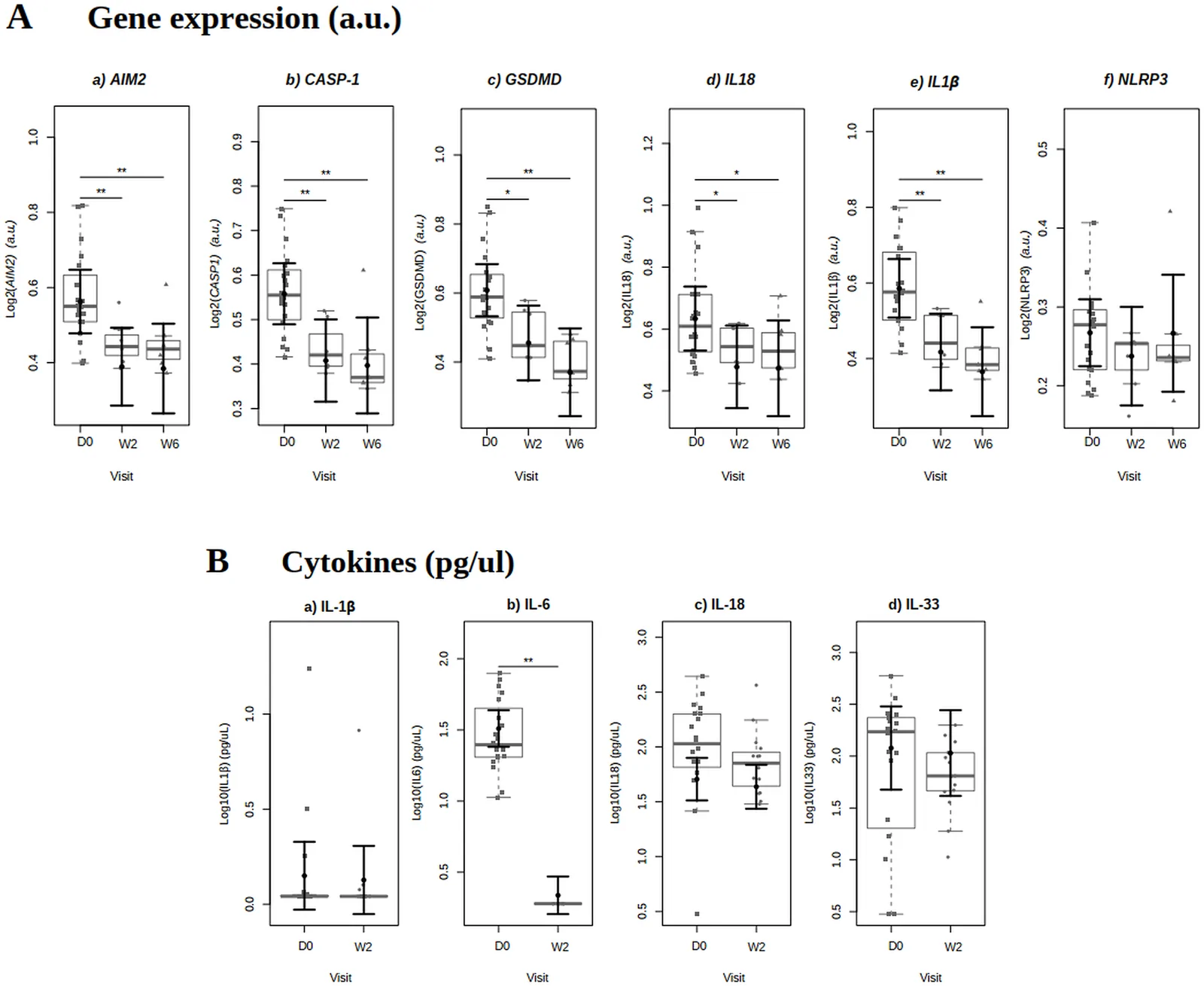
Longitudinal comparison of whole-blood inflammasome-related gene expression **(A)** and plasma cytokine levels **(B)** during follow-up in the TB–HIV group. **(A)** AIM2 **(a)**, CASP1 **(b)**, GSDMD **(c)**, IL18 **(d)**, IL1B **(e)**, and NLRP3 **(f)**. **(B)** Plasma levels of IL-1β **(a)**, IL-6 **(b)**, IL-18 **(c)**, and IL-33 **(d)**. Inflammasome-related gene expression was assessed at D0, W2, and W6, whereas plasma cytokine levels were measured only at D0 and W2. Boxplots show the distribution of log-transformed values within each time point (D0, W2, W6). Boxes represent the interquartile range (IQR), and the central horizontal bars represent the sample medians. Highlighted black vertical lines represent the 95% confidence intervals (95% CI) of the estimated marginal mean values derived from linear mixed-effects models adjusted for age, gender, skin color, schooling, and CD4+ T-cell count, including a random intercept for participant ID to account for repeated measures (central circle indicates the marginal mean). Gene expression values were transformed using *Log_2_(x+2)-1*, and cytokine concentrations were transformed using *Log_10_(x+10)-1*. Pairwise contrasts between marginal means across time points were estimated with Tukey’s adjustment. *P < 0.05; **P < 0.01.

## 4 Discussion

This study investigated the complex interaction between inflammasome activation and cytokine profiles in TB–HIV individuals, the HIV group, the TB group, and healthy controls. Our results provide evidence of a coordinated transcriptional upregulation of inflammasome-related pathways in TB-HIV individuals, highlighting increased expression of AIM2, CASP1, GSDMD, IL-1β, and IL-18 as well as elevated levels of circulating pro-inflammatory cytokines. The increased AIM2 expression observed in the absence of detectable changes in NLRP3 transcript abundance suggests that AIM2-related pathways warrant further investigation in TB–HIV coinfection (Fan et al., 2022). Furthermore, we observed significant changes in these transcriptional markers following the initiation of anti-TB therapy and ART. Together, these findings highlight the importance of further investigating innate inflammatory pathways in TB-HIV coinfection.

The exacerbated inflammatory state observed in this study among TB-HIV-coinfected individuals is consistent with the literature, which highlights dysregulated inflammation in TB-HIV coinfection (Lai et al., 2015; Tan et al., 2015; Dumitrescu et al., 2026). Critically, the absence of significant changes in NLRP3 expression, both at baseline and longitudinally, points towards a transcriptional signature consistent with enhanced inflammasome-related activity, particularly involving AIM2, with no evidence of significant NLRP3 transcript upregulation. Importantly, unchanged NLRP3 mRNA expression does not exclude post-transcriptional or post-translational regulation or functional NLRP3 inflammasome activation, which was not directly assessed in this study. This finding is particularly intriguing, as NLRP3 is a well-studied inflammasome sensor implicated in various infectious and inflammatory diseases (Man and Kanneganti, 2015; Xiao et al., 2026). Our data suggest that AIM2-related pathways may be involved in the inflammatory profile observed in TB-HIV co-infection, possibly in association with the detection of bacterial and/or viral DNA. However, these associations do not establish causality. The increased expression of AIM2 and its strong correlations with downstream inflammasome-related molecules, including CASP-1, GSDMD, IL-1β, and IL-18, suggest that the AIM2 cascade pathway may play a significant role in the inflammatory responses observed in coinfected individuals.

The AIM2 receptor, an inflammasome sensor implicated in host defense against pathogens such as *Mycobacterium tuberculosis* and HIV, is composed of a C-terminal HIN-200 domain and an N-terminal pyrin domain (PYD) and acts as a cytosolic sensor of microbial and host-derived double-stranded DNA, promoting inflammasome assembly, caspase-1 activation, pyroptosis, and the subsequent release of IL-1β and IL-18 (Lamkanfi and Dixit, 2009; Wawrocki and Druszczynska, 2017; Theobald et al., 2024). In the context of TB, it has already been described that macrophages infected with *Mycobacterium tuberculosis* activate the AIM2 inflammasome in response to mycobacterial DNA, promoting IL-1β and IL-8 production and contributing to host defense mechanisms (Law et al., 1996). However, excessive activation may also enhance alveolar inflammation and tissue damage through increased production of pro-inflammatory cytokines such as IL-1β and TNF-α (Briken et al., 2013). In early infection, AIM2 binds to HIV dsDNA and triggers acute inflammation and pyroptosis (Ekabe et al., 2021). AIM2 inflammasome has been associated with increased production of pro-inflammatory cytokines, such as IL-1β and IL-18, in HIV patients (Feria et al., 2018; Phoswa et al., 2023). Thus, the increased AIM2 expression and associated transcriptional pattern observed in TB–HIV coinfection may reflect the convergence of pathogen-associated and host-derived danger signals, potentially contributing to sustained inflammasome-related signaling and exacerbated inflammatory responses (Lamkanfi and Dixit, 2009; Yaseen et al., 2025). One possible mechanism underlying this process is pyroptotic cell death, which leads to the release of intracellular components, including mitochondrial DNA (mtDNA), into the systemic circulation (Bergsbaken et al., 2009). Extracellular mtDNA may subsequently activate AIM2 inflammasomes, thereby amplifying the inflammatory cascade (Nakahira et al., 2011).

Longitudinal analyses revealed significant reduction in the expression of AIM2, CASP1, GSDMD, IL18, and IL1β during clinical follow-up. Notably, inflammasome-related gene expression and plasma IL-6 levels were significantly reduced at W2, following two weeks of anti-TB therapy and immediately before ART initiation, compared with baseline. Therefore, the changes observed between D0 and W2 occurred during anti-TB therapy in the absence of ART exposure and are consistent with an early anti-TB treatment-associated effect. At W6, participants had received six weeks of anti-TB therapy and four weeks of ART; consequently, the specific contribution of ART to the changes observed at this time point cannot be distinguished from the ongoing effects of anti-TB treatment. One possible explanation for the observed reduction in AIM2-related transcript levels is a decrease in pathogen-associated antigenic burden during therapy; however, this mechanism was not directly assessed in the present study. Overall, these findings suggest treatment-associated modulation of the inflammasome-related transcriptional profile during clinical follow-up. These findings are particularly relevant in the context of immune reconstitution inflammatory syndrome (IRIS), a condition characterized by exacerbated inflammatory responses after ART initiation, in which increased innate immune activation and elevated IL-1β and IL-18 levels have been associated with IRIS development (Meintjes et al., 2008; Lai et al., 2016; Tan et al., 2016).

In a previous study, we reported an increased risk of TB-HIV/IRIS among individuals carrying specific AIM2 single-nucleotide polymorphisms (SNPs) (Sá et al., 2022). In contrast, the present study found no significant differences in AIM2 mRNA expression levels between TB-HIV individuals who developed IRIS and those who remained IRIS-free. Baseline plasma IL-1β levels were lower among individuals who subsequently developed IRIS; however, this exploratory finding should be interpreted cautiously because only four IRIS cases were included in this study. Accordingly, all IRIS-related analyses in the present study should be regarded as exploratory. This information is consistent with previous reports relating to this cytokine in IRIS pathogenesis. IL-1β plays dual and context-dependent roles in immune responses, contributing both to antimicrobial host defense and immunopathology. While IL-1β promotes pathogen control through the induction of antimicrobial mechanisms and T cells expansion and differentiation, persistent production during chronic infection may drive excessive inflammation and tissue damage, ultimately contributing to disease severity and pathological manifestations (Keyel, 2014; Rammaert et al., 2023). It is imperative to further contextualize this hypothesis-generating finding by acknowledging both the statistical and analytical limitations inherent to our IL-1β/IRIS sub-analysis. From a statistical standpoint, the inference is severely underpowered due to the restricted number of incident IRIS cases (n=4), rendering the estimated effect highly susceptible to Type I error. Consequently, the lower baseline IL-1β levels observed among IRIS cases should not be interpreted as evidence of a protective effect and requires rigorous validation in larger, adequately powered cohorts utilizing ultra-sensitive quantification platforms.

The elevated plasma levels of IL-6, IL-18, and IL-33 at the baseline further support the presence of an intensified inflammatory environment in TB-HIV individuals. Furthermore, although gene expression and plasma cytokine levels offer valuable insights, additional functional studies are required to fully understand the activity of inflammasome pathways. A more in-depth analysis of the PYHIN family receptors and their pathways is required in the case of TB-HIV co-infection.

Further exploratory support for a distinct inflammasome-associated inflammatory profile in TB–HIV co-infection was provided by the PCA analysis. The observed clustering pattern was primarily driven by variables related to AIM2, CASP1, GSDMD, and IL18, which was consistent with the overall molecular profile observed in the other analyses. As an exploratory dimensionality-reduction analysis, PCA was used to visualize overall patterns in the molecular data and should not be interpreted as independent evidence of a distinct functional inflammasome pathway.

The main limitation of this study was the small sample size, particularly the low number of TB-HIV/IRIS cases included, and these analyses should be considered exploratory. This was likely due to the reduced recruitment of TB-HIV co-infected individuals during the study period, despite Rio de Janeiro being a region where TB is endemic. The lower-than-expected enrollment may have been influenced by the implementation of universal free early access to antiretroviral therapy by the Brazilian Ministry of Health, independent of CD4+ T-cell counts or clinical status, which likely reduced the number of severely immunosuppressed individuals at risk of developing IRIS.

Severe additional limitations should be considered. The study primarily assessed transcript abundance and circulating cytokine concentrations and did not directly evaluate inflammasome assembly, caspase-1 activation, GSDMD cleavage, or pyroptotic cell death. Finally, PCA was exploratory and require independent validation. Larger cohorts and functional studies are needed to validate these transcriptional findings and clarify the contribution of AIM2- and NLRP3-related pathways to TB-HIV coinfection and IRIS.

In conclusion, TB–HIV coinfection was associated with a distinct inflammasome-related transcriptional and inflammatory profile characterized by increased AIM2, CASP1, GSDMD, IL1B, and IL18 expression and elevated circulating pro-inflammatory cytokines. These changes decreased during anti-TB therapy and subsequent ART, suggesting treatment-associated modulation of this inflammatory profile. Although NLRP3 transcript abundance did not differ significantly across groups or time points, the present findings do not exclude regulation of NLRP3 at the protein or functional level. Further studies incorporating direct measurements of inflammasome assembly, caspase-1 activation, GSDMD cleavage, and pyroptosis are required to clarify the contribution of AIM2-related pathways to TB–HIV coinfection and IRIS.

## Data Availability

The datasets for this article are not publicly available due to concerns regarding participant confidentiality and ethical restrictions related to human subject data. Requests to access the de-identified datasets should be directed to the corresponding authors, Mariza Gonçalves Morgado (mmorgado@ioc.fiocruz.br) and Nathalia Beatriz Ramos de Sá (nathalia.beatriz2008@gmail.com), and will be considered in accordance with applicable ethical and institutional requirements.
