# Supplementary material for: Inflammasome-associated immune dysregulation in TB–HIV coinfection
Source: Front Cell Infect Microbiol. 2026 Sep 30;16:1961755. doi: 10.3389/fcimb.2026.1961755 (PMC13661482; doi:10.3389/fcimb.2026.1961755)
Supplement: Supplementary file 1 [file Table1.docx]

**Supplementary Material**

**Table S1: TaqMan Gene Expression Assays used for the quantification of inflammasome-related genes.**

| Gene Symbol | Gene  Name | Assay ID* | Chromosome  Location | Amplicon Length |
| --- | --- | --- | --- | --- |
| AIM2 | Absent in melanoma 2 | Hs00915710_m1 | 1 | 106 |
| CASP1 | Caspase-1 | Hs00354836_m1 | 11 | 76 |
| GSDMD | Gasdermin D | Hs00986748_g1 | 8 | 63 |
| IL18 | Interleukin 18 | Hs01038788_m1 | 11 | 115 |
| IL-1β | Interleukin 1 beta | Hs01555410_m1 | 2 | 91 |
| NLRP3 | NLR family pyrin domain containing 3 | Hs00918082_m1 | 1 | 84 |

*Thermo Fisher Scientific assay identification.
